# A commentary of “Rhythmic cilia changes support SCN neuron coherence in circadian clock”: Top 10 Scientific Advances of 2023, China

**DOI:** 10.1016/j.fmre.2024.03.021

**Published:** 2024-04-02

**Authors:** Wei Li, Guangshuo Ou

**Affiliations:** a School of Medicine, Tsinghua University, Beijing 100084, China; b Tsinghua-Peking Center for Life Sciences, Beijing Frontier Research Center for Biological Structure, McGovern Institute for Brain Research, State Key Laboratory of Membrane Biology, School of Life Sciences and MOE Key Laboratory for Protein Science, Tsinghua University, Beijing 100084, China

Primary cilia, microtubule-based cellular protrusions found on the surface of nearly all eukaryotic cells, serve as crucial signaling hubs [1]. These antenna-like organelles play a pivotal role in perceiving environmental stimuli, thereby orchestrating cellular behavior and fate. Primary cilia are essential for various signaling pathways, including the Sonic Hedgehog (SHH) pathway. Ciliary dysfunction can affect multiple organ systems and manifest as a spectrum of genetic disorders collectively termed as ciliopathies. However, until recently, their involvement in circadian rhythms was unknown.

Circadian rhythms are regulated by an internal circadian clock that governs vital metabolism, physiology, and behavior, mostly via a roughly 24-hour sleep-wake cycle [2]. Maintaining a stable circadian rhythm is essential for overall health, including hormone secretion, metabolism, and gene expression. The mammalian suprachiasmatic nucleus (SCN) is the principal circadian clock that synchronizes daily rhythms of behavior and physiology to the external environment. Disruption of circadian rhythms, such as sleep disturbances, can lead to a variety of health issues, including sleep disorders, psychiatric disorders, metabolic disorders, and increased risk of cancer and cardiovascular diseases. Understanding circadian rhythms is thus crucial for improving health and overall well-being, as evidenced by the 2017 Nobel Prize in Physiology or Medicine for the discovery of the molecular mechanisms underlying circadian rhythms.

Teams led by Drs. Hui-Yan Li and Xue-Min Zhang have uncovered a critical mechanism governing circadian rhythms within the mouse brain's hypothalamic SCN. They found that the SHH signaling pathway, dependent on primary cilia, plays a pivotal role in regulating these rhythms [3]. This discovery sheds light on the intricate regulation of circadian rhythms by primary cilia and SHH signaling (Fig. 1). This study unveiled that a substantial portion of SCN neurons possess primary cilia. Intriguingly, the length and abundance of these cilia in neurons expressing the neuropeptide neuromedin S (NMS) oscillated in accordance with the daily light-dark cycle, indicating their role in circadian rhythms. Conditional mutation of intraflagellar transport (IFT) genes crucial for ciliogenesis in NMS neurons resulted in reduced intercellular coupling within the SCN and accelerated phase shifts of the internal clock under conditions mimicking jet lag. Moreover, this study demonstrated the circadian rhythmicity of SHH signaling in NMS neurons, with the expression of the SHH gene being pivotal for synchronizing the SCN network. Notably, both genetic and pharmaceutical inhibition of SHH signaling phenocopied the deficiencies observed in IFT mutants. Strikingly, mice lacking SHH signaling adapted more swiftly to altered light cycles, revealing a novel function of primary cilia and SHH signaling in circadian regulation. Acknowledged as one of the “Top 10 Scientific Advances of 2023, China”, this study identified primary cilia as an unrecognized regulator of circadian rhythms, enhancing our comprehension of how the internal clock maintains robustness against environmental disruptions. These findings hold significant implications for human health, opening avenues for therapeutic interventions targeting SHH signaling and other cilia-related pathways to address circadian rhythm disorders.

**Fig. 1:**
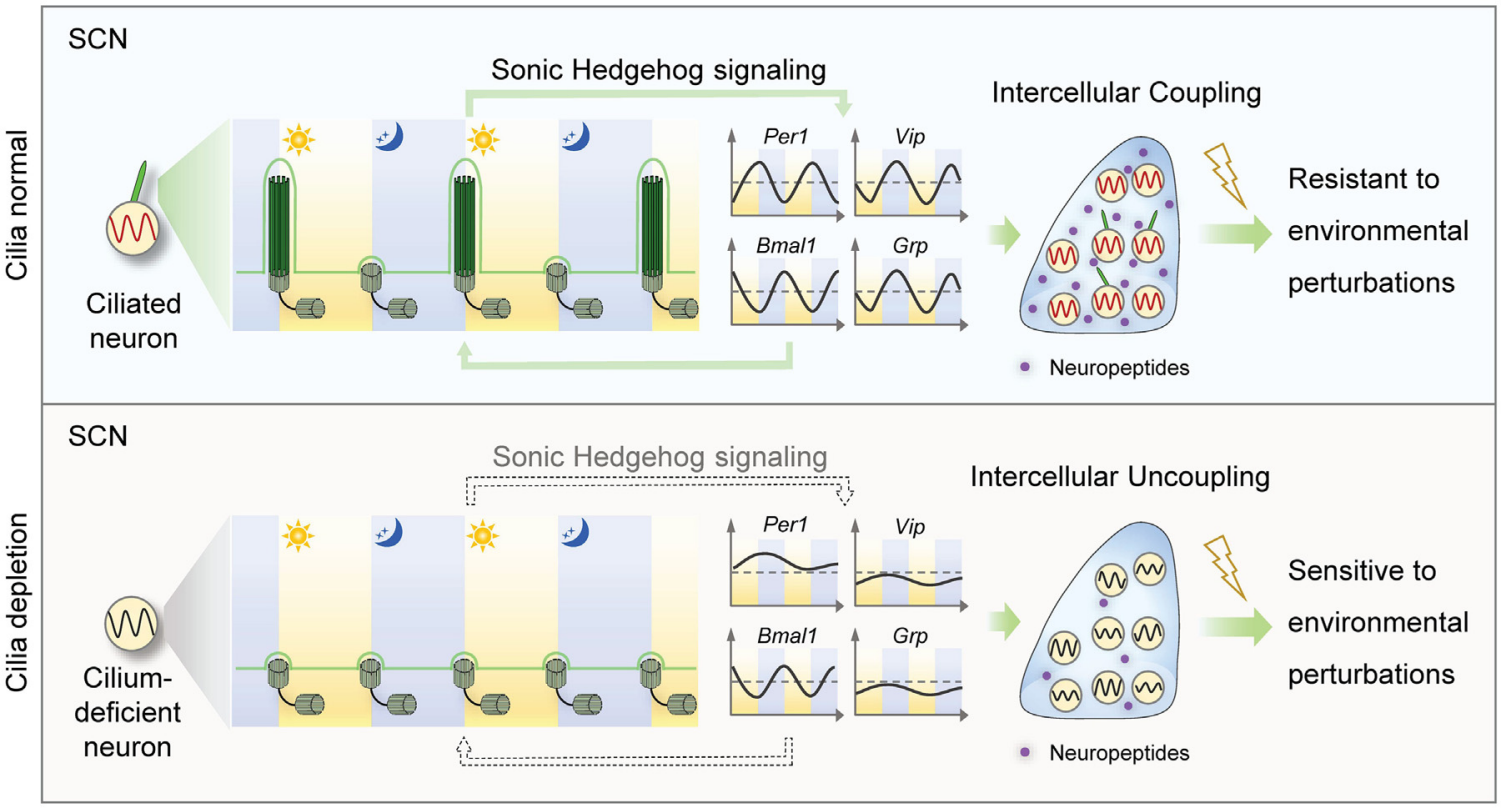
**Summary diagram showing primary cilia in SCN neurons and downstream Hedgehog signaling as critical regulatory mechanisms promoting interneuronal coupling, thereby maintaining SCN network synchrony and circadian rhythms**[3].

In China, research on circadian biology has experienced notable growth, contributing to our understanding of circadian rhythms across molecular, cellular, and behavioral domains. While these studies concentrate on identifying molecules governing circadian clocks and the regulatory mechanisms orchestrating rhythmic processes, there remains considerable uncharted territory concerning circadian rhythms within intracellular organelles and their functional implications. Tu et al.'s discovery of an unforeseen functional role of the primary cilium—a sensory organelle—and cilia-mediated SHH signaling in the internal circadian clock represents a significant breakthrough. These findings highlight the intricacies of the mechanisms underpinning circadian rhythms and pave the way for investigating the connections between ciliary function and circadian health. Future research in circadian rhythm holds promise for uncovering additional circadian organelles and unraveling the intricate molecular interactions governing the circadian clock. Such endeavors could yield significant advancements, including the identification of novel cilia-related proteins and signaling pathways implicated in circadian regulation. Moreover, the emergence of new therapeutic strategies targeting these pathways may offer avenues for restoring circadian rhythms in individuals afflicted with circadian disorders.

Equally captivating is the research of cilium biology in China. In addition to the significant contributions of China-based research groups to the traditional realms of cilium biology, this exploration into the novel ciliary function within circadian rhythms heralds a new direction and inspires cell biologists to venture beyond established fields. Cilia have long been regarded as one of the simplest organelles, with their molecular components extensively documented and their fine architecture resolved at near-atomic resolution. However, this recent work suggests that our comprehension of ciliary function and regulation may only scratch the surface. This notion finds support in a recent study uncovering how RNA editing restricts hyperactive ciliary kinases [4], hinting at a previously unrecognized feedback loop from protein activity to the mRNA encoding it. In these two studies, delving into ciliary behavior and function in SCN neurons and analyzing the RNA editome in ciliary mutants respectively serve as driving forces for advancing cilia research, emphasizing the necessity for a paradigm shift to invigorate this classic field. Building upon this groundwork, a comprehensive examination of ciliary behavior and function across various cell types within a living organism, coupled with the development of essential tools, may shed light on new facets of ciliary physiology. Conversely, the recent AlphaMissense platform predicts that over 23 million missense mutations in the human proteome could be pathological [5], with many linked to ciliopathies. Hence, precision genomic medicine targeting cilia must be refined to the level from individual genes to each nucleotide. Alterations at each amino acid within a ciliary protein could yield distinct impacts, necessitating unique interventional strategies. This underscores the burgeoning field of functional residuomics of cilia, which furnishes residue-specific functional insights into the proteomic landscape of cilia.

## Declaration of competing interest

The authors declare that they have no conflicts of interest in this work.
